# The challenging diagnosis and follow-up of skull base osteomyelitis in clinical practice

**DOI:** 10.1007/s00405-020-06576-6

**Published:** 2021-01-28

**Authors:** Alice B. Auinger, Valerie Dahm, Isabella Stanisz, Ursula Schwarz-Nemec, Christoph Arnoldner

**Affiliations:** 1 Department of Otorhinolaryngology, Medical University of Vienna, Waehringer Guertel 18-20, 1090 Vienna, Austria; 2 Department of Biomedical Imaging and Image-Guided Therapy, Medical University of Vienna, Waehringer Guertel 18-20, 1090 Vienna, Austria

**Keywords:** Skull base osteomyelitis, Osteomyelitis, Malignant otitis externa, Necrotizing otitis externa, Cranial nerve palsy

## Abstract

**Purpose:**

The disease activity of skull base osteomyelitis can be challenging to assess by means of conventional imaging methods and renders monitoring of the disease difficult, especially in areas with restricted access to nuclear medicine imaging. Here, we provide clinically relevant data on the management of skull base osteomyelitis including assessment, treatment, and follow-up strategies with regards to the role of imaging.

**Method:**

A chart review was performed including 30 patients treated for SBO from 1993 to 2015. Clinical findings, treatment procedures, and complication rates were assessed. Special attention was paid to imaging procedures.

**Results:**

The overall mortality rate was 36.7% and increased to 45% when cranial nerve palsies were present. An initial computed tomography (CT) scan was performed in all patients, MRI in 60% and nuclear imaging in 33%. CT scans failed to detect progression or regression in up to 80% after four to nine months. MRI examinations could reveal changes at a higher rate compared to CT. Nuclear medicine functional imaging was most likely to assess disease activity.

**Conclusion:**

A combination of different imaging modalities is recommended for diagnosing SBO. For the follow-up, MRI is preferable to CT as changes can be detected more readily with MRI. If available, nuclear medicine imaging should guide the decision of treatment discontinuation.

## Introduction

Skull base osteomyelitis (SBO) is a rare but severe and life-threatening disease. It mainly occurs secondary to otogenic and sinugenic infections via contiguous spread. Pain is often the only symptom and typical clinical findings associated with external otitis or otitis media are often missing [1]. Diabetes, cardiovascular diseases, and immunosuppression are known to be the most common comorbidities in SBO patients [2–4]. Despite improved diagnostic modalities and the use of modern antibiotic agents, mortality and morbidity rates remain high. Data of the Californian population revealed a median incidence of otogenic SBO of 75.5 cases per year [5]. Due to an aging and subsequently increasingly multimorbid population, SBO is on the rise [6]. Therefore, it is important to raise awareness for this disease as limited evidence of diagnostic, therapeutic, and follow-up strategies render management difficult [7].

Finding the correct diagnosis is the first challenge when faced with the question of SBO. No single imaging modality is able to detect the extent of this disease. Active inflammation is difficult to distinguish from resolving abnormalities in conventional imaging modalities. Nuclear medicine functional imaging methods alone or a combination with high-resolution imaging for anatomy such as single-photon emission computed tomography (SPECT) or positron emission tomography (PET) with computed tomography (CT) or magnetic resonance imaging (MRI) are recommended but not often available [8–10].

Once the diagnosis of SBO is made, the next difficulty is finding the correct therapy and consecutively deciding on the necessary duration of treatment. Pseudomonas aeruginosa has been reported to be the main pathogen in 50–98% of SBO [3, 11, 12]. However, in up to a third of patients no pathogen can be isolated [13, 14]. Most patients are treated with topical or oral antibiotics when they present at primary care physicians. In cases where recovery has failed, pre-treatment can lead to negative cultures. Surgery was once advocated as the treatment method of choice [15]. Long-term intravenously administered antibiotic agents are indicated when the diagnosis is made with a median of 3.5 agents used in a patient [2, 16]. Hyperbaric oxygen therapy might be an effective adjuvant in advanced or treatment-refractory SBO [17].

Further, there is no consensus on the preferred imaging modality to assess disease progress or relapse as well as treatment efficacy. Primary or secondary care hospitals do not have the ability to assess disease activity with nuclear medicine imaging. Most clinicians have to rely on conventional imaging modalities such as MRI and CT. In this study, patients with clinical signs of SBO, who were treated in a tertiary care hospital, were evaluated. Apart from outcome parameters such as cranial nerve function and mortality rates, special attention was paid to conventional follow-up imaging. The main aim of the present study was to evaluate the outcome of patients with SBO and to assess the best possibility for conventional follow-up imaging methods.

## Materials and methods

A chart review was performed including patients with clinically diagnosed SBO, who were treated from January 1993 to December 2015 in a tertiary care hospital. The patients’ database was searched for the diagnosis “malignant otitis externa”, “skull base osteomyelitis”, and “petrositis”. A total of 30 patients met inclusion criteria, which were clinical signs of SBO such as inflammation of the ear and/or facial nerve palsy and/or erosion of the temporal bone on any imaging method. An otogenic cause was assumed in 80% (*n* = 24), a sinugenic cause in 3.3% (*n* = 1) and in 16.7% (*n* = 5) the origin of the infection remained unclear.

Based on medical records, hospitalization time, clinical symptoms including complications such as cranial nerve palsies, comorbidities, culture results of ear swabs, treatment, and the mortality rate were assessed. If available, histopathologic reports of biopsies and laboratory results including leucocyte count, creatinine clearance, and C-reactive protein (CRP) on admission and discharge were evaluated.

The role of high-resolution CT in diagnosing SBO and during follow-up procedures has been a matter of discussion. Therefore, special attention was paid to imaging reports performed for diagnosis and during follow-up of SBO. Imaging modalities included CT, MRI, and nuclear medicine functional imaging methods. Nuclear medicine imaging methods such as scintigraphy, single-photon emission tomography (SPECT) or positron emission tomography (PET) were performed to assess functional aspects rather than anatomical structures. Tc-99m MDP detects osteoblastic activity and was the most frequently used tracer in this study. Other tracers used in the current study were Tc-99m labelled leukocyte antibodies, which accumulate in infectious areas such as found in SBO and beta-emitting tracers like 2-Fluor-2-Desoxy-Glucose (FDG), which detect increased metabolism as in infectious or malignant diseases. Four CT scans were performed for CT-guided biopsy and abscess drainage and were not included in the analysis.

During hospitalization, all patients were primarily treated with broad-spectrum intravenous antibiotics until sensitivity patterns of culture results were available. Blood glucose levels were optimized if necessary. Cases which could not be connected to SBO where classified as death due to other reasons. The study was approved by the local ethics committee (EK 1286/2016). Due to the retrospective nature of this study, no informed consent of included patients was required.

## Results

### Demographic results and hospitalization time

Of 30 included patients (10 females, 20 males), 50% (*n* = 15) were referred from previous in-patient treatment at other hospitals, and 50% (*n* = 15) were referred from general practitioners. The mean age on admission was 73.1 ± 13.5 years. The right temporal bone was affected by 36.7% (*n* = 11) and the left side in 63.3% (*n* = 19). A second admission was necessary for 33.3% (*n* = 10). Some patients required inpatient treatment for a third or fourth time (see Table 1 for demographic and clinical characteristics of all included patients). Patients were admitted 1.4 ± 0.7 times on average with a mean hospitalization time of 50.4 ± 53.1 days.

**Table 1:** Demographic details of included subjects

ID	Sex	Age	CNP	Side	HT	Admissions	Survival	Comorbidities
1	M	71	n	r	102	3	Y	DM, H
2	F	70	VII	l	80	2	N	DM
3	M	36	n	l	20	1	Y	n
4	M	87	n	r	38	2	N^a^	H
5	M	86	VII	l	233	4	N	DM, CKI
6	M	86	VII	l	30	2	Y	n
7	M	51	n	l	45	1	N^a^	DM, PAVK
8	M	83	n	l	29	1	N	DM
9	M	68	n	l	15	1	Y	DM
10	F	79	VII	l	34	1	N	DM, H
11	F	79	III, VI, V, VII	l	145	1	N	H, Polyarthritis
12	F	79	VII	l	17	1	N	DM, H, CAVK
13	M	85	VI	l	7	1	N	DM, CAVK
14	M	87	n	r	9	1	Y	DM, H, CAVK
15	M	63	VII	r	6	1	N^a^	DM, H, CAVK, KT
16	M	72	VII	l	19	1	N	H, KT
17^†^	M	81	II, VII, IX, X	l	–	–	N	DM, H, cAVK
18	F	58	VII, X, XI	r	107	2	N	H, LY
19	F	77	n	r	50	1	Y	DM
20	M	71	VII	l	17	1	Y	DM, H
21	M	93	VI	l	44	2	Y	cAVK
22	F	59	VII	r	19	1	Y	DM, cAVK
23	M	60	VII, IX, XII	l	25	1	Y	DM, H
24	F	88	n	r	16	1	N^b^	DM, H
25	M	70	VII	r	111	2	Y	DM, cAVK
26	F	77	n	r	3	1	N	LY
27	F	72	VIII	l	156	1	Y	DM, LY
28	M	89	V, VII, IX, XII	r	62	1	Y	DM
29	M	63	VI, X, XII	l	28	2	N^a^	DM
30	M	49	VI	l	44	1	Y	DM, H

^†^Patient No. 17 was hospitalized at another hospital but second opinion was requested—complete data were not available; *m* male, *f* female, *r* right, *l* left, *HT* hospitalization time in days, *y* yes, *n* no, C*K*I chronic kidney disease, *DM* diabetes mellitus, *H* arterial hypertension, *KT* kidney transplantation, *LY* lymphoma, *PAD/CAD* peripheral arterial/coronary artery disease, *N*^a^ death not associated with SBO, *N*^b^ reason for death unknown, *Admissions* number of admissions for inpatient treatment

### Clinical findings, symptoms and comorbidities

Clinical symptoms and findings on admission were distributed as follows (multiple symptoms per patient are listed): otalgia and headache 76.6% (*n* = 23) followed by otorrhea 40.0% (*n* = 12), inflammation of the external ear canal 36.7%, (*n* = 11), granulation tissue of the external ear canal 26.7% (*n* = 8), and perforation of the tympanic membrane 6.7% (*n* = 2).

The most common comorbidity was diabetes (73.3%, *n* = 22). Arterial hypertension was observed in 46.7% (*n* = 14), peripheral vascular disease in 20.0% (*n* = 6), and 26.7% (*n* = 8) of included patients were immunosuppressed due to kidney transplant, lymphoma or polyarthritis.

### Diagnostic results

#### Laboratory results

Laboratory results of the following parameters on admission were assessed: leukocyte count of 8.3 ± 3.0, CRP of 4.8 ± 5.9, and creatinine of 1.2 ± 0.4. At patients’ discharge, the leukocyte count was 7.5 ± 3.7, CRP 1.8 ± 3.2, and creatinine 1.8 ± 0.5. The glycated hemoglobin (HbA1c) was available from 10 patients and was 8.7 ± 2.3.

#### Microbiologic results

Microbiologic swabs verified pathogens in 80.0% (*n* = 24) on the first admission, whereas no pathogen was detected in 20.0% (*n* = 6) of all patients; multiple pathogens were observed in 36.7% (*n* = 11) and a single one in 43.3% (*n* = 13). By far the most frequently detected bacterium was Pseudomonas aeruginosa in 75.0% (*n* = 18), followed by Staphylococcus aureus in 29.2% (*n* = 7) and Candida in 8.3% (*n* = 2). Further pathogens were Corynebacterium (12.5%, *n* = 3), Staphylococcus epidermidis and Coagulase-negative Staphylococci in 8.3% (*n* = 2 each), and Escherichia coli, Streptococcus anginosos, Aspergillus fumigatus/nidulans, Citrobacter freundii, oropharyngeal bacteria, and Propionibacterium acnes in 4.2% (*n* = 1 each). 3-MDRGN Pseudomonas aeruginosa appeared in 8.3% (*n* = 2).

#### Imaging methods

In total, 70 CT examinations including first and follow-up scans were performed. An initial CT scan was performed in all included patients (*n* = 30). Multiple findings on one scan could be commonly assessed but the most common pathologies were soft tissue changes in 53% (*n* = 16) and bone erosion in 43% (*n* = 11). A second CT scan was performed in 70.0% (*n* = 21) of patients with a mean of 55.5 ± 52.7 days between the first and second scan. Disease progression was found in 57.0% (*n* = 12) of follow-up scans, regression was seen in 14.0% (*n* = 3) and 29.0% (*n* = 6) showed no change. A third (*n* = 10), fourth (*n* = 8) and fifth (*n* = 2) CT scan for follow-up was performed in several patients, but most of the scans revealed no change of findings. Details on results are shown in Table 2. CTs performed for nuclear medicine imaging modalities were not included.

**Table 2:** First and follow-up imaging with CT and MRI

	CT1 (30)	CT2 (21)	CT3 (10)	CT4 (8)	CT5 (2)
Soft tissue changes	53% (16)	55% (11)	50% (5)	38% (3)	100% (2)
Bone erosion	43% (13)	55% (11)	60% (6)	50% (4)	
Nerve erosion	7% (2)	–			
Cholesteatoma	3% (1)	–			
Abscess	3% (1)	10% (2)	10% (1)		
Post-surgery defect		5% (1)	10% (1)		
No pathology	13% (4)			13% (1)	
Interval^a^		55.5 ± 52.7	67.6 ± 72.2	155.1 ± 311.9	44.5 ± 41.7
Dynamics to last CT					
Progress		57% (12)	10% (1)	13% (1)	50% (1)
No change		29% (6)	80% (8)	62% (5)	50% (1)
Regression		14% (3)	0% (0)	25% (2)	
			10% (1)		

	MRI1 (18)	MRI2 (12)	MRI3 (10)	MRI4 (6)	MRI5 (4)	MRI6 (3)
Soft tissue changes	33% (6)	33% (4)	20% (2)	33% (2)	25% (1)	33% (1)
Bone erosion	56% (10)	67% (8)	60% (6)	83% (5)	75% (3)	67% (2)
Nerve erosion	56% (10)	8% (1)	20% (2)	33% (2)	50% (2)	67% (2)
Meningitis	6% (1)		10% (1)	17% (1)	25% (1)	33% (1)
Thrombosis of ICA		8% (1)	10% (1)	17% (1)	25% (1)	33% (1)
Abscess			10% (1)	17% (1)	25% (1)	
No pathology	11% (2)					
Interval^a^		82.4 ± 77.1	32.2 ± 21.5	40.3 ± 27.7	51.5 ± 42.2	93.7 ± 78.4
Dynamics to last MRI						
Progression		50% (6)	40% (4)	33% (2)	75% (3)	0% (0)
No change		25% (3)	20% (2)	50% (3)	25% (1)	67% (2)
Regression		25% (3)	40% (4)	17% (1)	0% (0)	33% (1)

Findings on CT and MRI scans according to reports given as percentages and numbers (brackets). Multiple findings on one scan possible

^a^Time in days between first and second, second and third, third and fourth scan and so forth. CT1 corresponds to the initial CT performed, CT2 to the second, CT3 to the third and so forth. MRI1 corresponds to the initial MRI performed, MRI2 to the second, MRI3 to the third and so forth. The CT and MRI scans of nuclear medicine functional imaging methods are not included

An initial MRI scan was performed in 60% (*n* = 18) of all included patients 43.8 ± 81.1 days after the initial CT scan; in two patients, the MRI was performed 7 and 52 days before the CT scan. Multiple findings on one scan could be assessed; the most common findings on the initial scan were bone erosion in 56% (*n* = 10), nerve erosion in 56% (*n* = 10), and soft tissue changes in 33% (*n* = 6). A second MRI scan was performed in 40% (*n* = 12) of patients with a mean of 82.4 ± 77.1 days between the first and second examination. In total, 57 MRI scans were performed on 18 patients including first and follow-up examinations. The main findings, the time between scans and imaging changes are displayed in Table 2. MRIs performed for nuclear medicine imaging modalities were not included.

A sum of an additional 14 nuclear medicine functional imaging examinations were performed in 33.3% (*n* = 10) of included patients. A positron emission tomography in combination with CT or MRI was performed once in three patients (two PET-CT scans and one PET-MRI), which revealed increased F18-FDG tracer uptake. A scintigraphy was performed 11 times; in eight of these in combination with single-photon emission computed tomography (SPECT), whereof four were performed in combination with a CT (SPECT-CT). Tracer which was used included Tc-99m MDP (*n* = 5), Tc-99m labelled leukocyte antibodies (*n* = 4), and Tc-88m DPD (*n* = 2). All but two scintigraphies revealed increased tracer uptake. One patient without increased tracer uptake showed a clear deterioration on MRI and the patient died in the course of the disease. The tracers used in the latter case were Tc-99m labelled leukocyte antibodies. Another patient without increased uptake of Tc-99m labelled leukocyte antibodies showed increased tracer uptake in the FDG-PET-CT as well as in the scintigraphy with SPECT and Tc-99 m MDP performed 1 month later.

### Treatment

#### Conservative treatment

All patients initially received broad-spectrum intravenous antibiotic therapy until swab results revealed the sensitivity of pathogens. Most commonly used antibiotic agents included ß-lactame antibiotics in 56.7% (*n *= 17), cephalosporines in 40% (*n* = 12), whereof third-generation cephalosporines were the main administered agent, epoxide antibiotics including fosfomycin in 30% (*n* = 9), fluoroquinolones in 23.3% (*n* = 7), and lincosamides in 23.3% (*n* = 7). Less frequently used antibiotics included carbapenems in 13.3% (*n* = 4), fusidic acid, macrolides, glycopeptides, trimethoprim/sulfonamides, lipopeptides, and aminoglycosides in 3.3% (*n* = 1) each. Patients with suspected fungal causes were treated with triazoles and echinocandines in 3.3% (*n* = 1) each. Patients received 2.6 different antibiotic agents on average.

#### Surgery

On the first admission, surgery was performed in 60.0% (*n* = 18) of included patients and comprised a mastoidectomy in 27.8% (*n* = 5), biopsies to rule out malignancy in 33.3% (*n* = 6), abscess drainage in 22.2% (*n* = 4), insertion of ventilation tubes in 16.7% (*n* = 3), one tympanoplasty (5.6%), one facial nerve decompression (5.6%), one functional endoscopic sinus surgery (5.6%) and removal of necrotic tissue in 16.7% (*n* = 3) with one skin grafting (5.6%). Histological results revealed granulation, necrotic or inflammatory tissue in all specimens. Out of 33.3% (*n* = 10) readmitted patients, four underwent a second surgery including two mastoidectomies, one VII decompression, and one removal of necrotic tissue with skin grafting.

### Mortality rate and complications

**Fig. 1:**
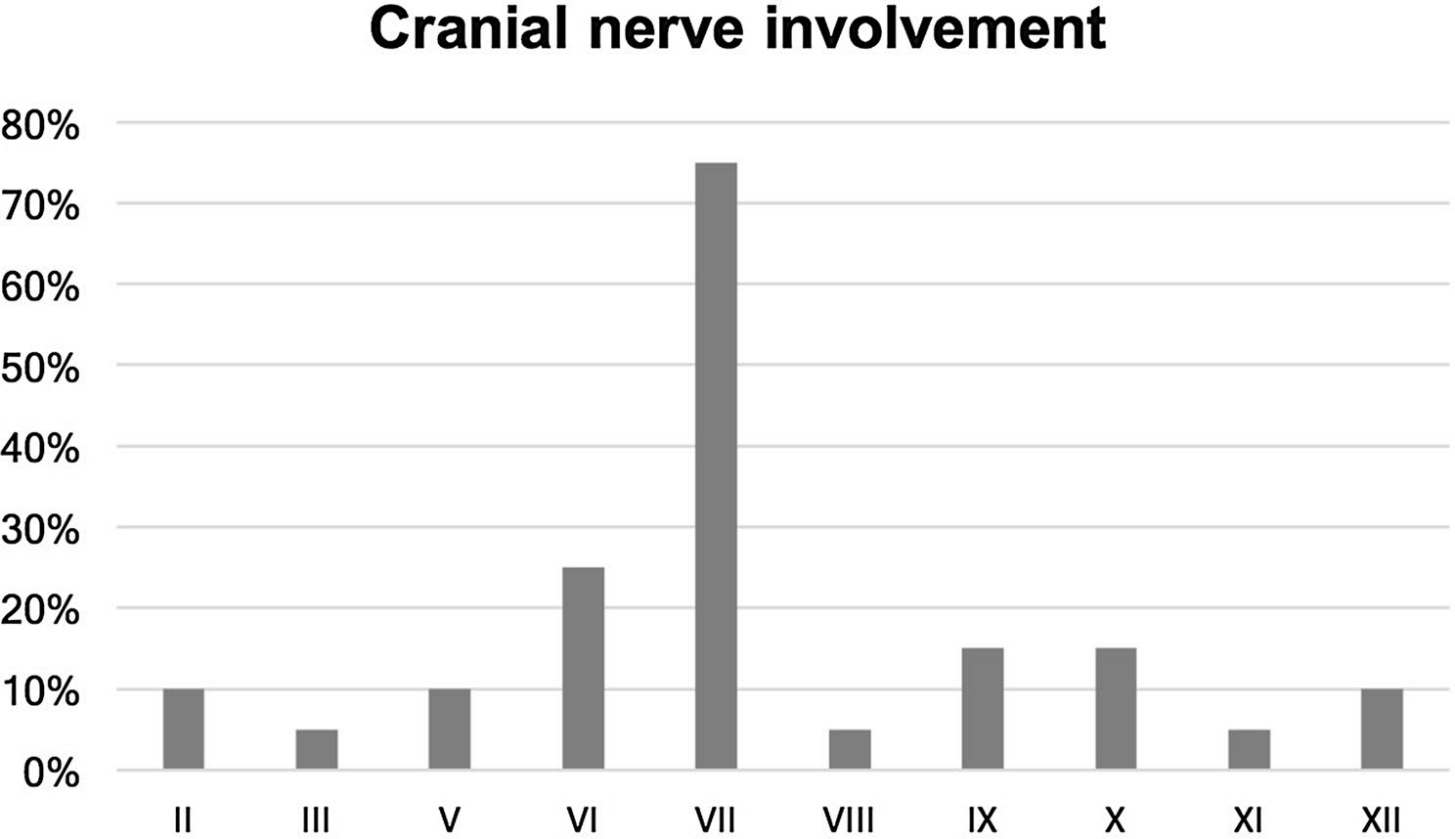
Rate of cranial nerve palsies in percentages; roman numerals indicate cranial nerves

The overall mortality rate was 53.3% (*n* = 16) of included patients, whereby 36.7% (*n* = 11) were directly associated with SBO and 16.7% (*n* = 5) died due to other reasons. Mean time between admission and death was 23.3 ± 18.7 weeks. On admission, cranial nerve palsy (CNP) was present in 66.7% (*n* = 20); single nerve involvement in 70.0% (*n* = 14) and multiple nerve palsies in 30.0% (*n* = 6). The most commonly affected nerve was the facial nerve in 75.0% (*n* = 15). No patient developed a new CNP during treatment. Figure 1 shows the distribution of affected cranial nerves. The mean hospitalization time of patients with CNP including all admissions was 61.3 ± 49.6 days. For patients without CNP the mean inpatient treatment duration was 43.6 ± 24.7 days. The mortality rate was 45% in 20 patients with CNP, whereas 20% in patients without CNP.

Of 46.7% (*n* = 14) patients who survived, reports of the last clinical follow-up examination 27 ± 34 months after initial admission were available in eight patients. Some dysfunction of the affected nerve remained in all patients who presented with CNP on first admission (*n* = 4, 50.0%).

## Discussion

We report on outcomes of patients with skull base osteomyelitis (SBO) with special attention to the usefulness of different imaging modalities for diagnosis and during follow-up.

In accordance with the literature, the majority of patients were immunosuppressed or had diabetes [16, 18]. SBO resulted mostly from otogenic causes with typical clinical findings on admission. Otalgia and headache have been reported to be the main symptoms as confirmed in this study [19, 20]. In some patients, the origin of the disease was not clear. Pain and mild changes in the otoscopy might occur weeks before the patient is seen by a physician or before CNP develops [19, 21, 22]. The affection of cranial nerves was high in this study. Single or multiple CNPs were present in more than two-thirds of patients on admission, predominantly the facial nerve. The presence of CNPs on admission has been stated to be a negative prognostic factor [2]. In contrast to the overall mortality rate of 36.7%, almost 50% of patients with CNPs died due to SBO compared to 20% of patients without CNPs. This underlines the importance of adequate diagnosis and aggressive treatment of this disease. Lower mortality rates of 8–19% were found by other authors [13, 14, 23]. However, the rate of CNPs and/or the involvement of multiple cranial nerves was also lower compared to our results (8–42% versus 66.7% in the current study). On the other hand, Sokolowski et al. found CNPs in 3 out of 7 included patients who all survived [19].

One of the biggest challenges when faced with the question of SBO is to rule out malignancy. Consequently, two-thirds of included patients underwent a surgical intervention such as biopsy, mastoidectomy or abscess drainage. Since long-term antibiotic therapy is necessary for SBO the most important step is isolating a pathogen. Pseudomonas aeruginosa is by far the most common pathogen and was verified in 80% of all patients in the current study. This was also observed by Lee et al. [2] and Prasad et al. [3], who found Pseudomonas aeruginosa in 74% and 80%, respectively. Some authors reported that Staphylococcus aureus is another frequent causative germ [3, 13, 16]. In this study, Staphylococcus aureus was found in approximately one third but mainly in combination with other bacteria identified through microbiological swabs.

The duration of antibiotic treatment has been reported to range from four weeks to several months; in fungal SBO even up to one year [2, 11, 14, 24, 25]. In the current study cohort, broad-spectrum intravenous antibiotics were administered initially followed by culture-directed treatment with an average length of 50 days. Increasing rates of ciprofloxacin-resistant Pseudomonas aeruginosa with no influence on mortality and morbidity have been reported [24]. In this study, 3-MDRGN Pseudomonas aeruginosa appeared in only two patients who were admitted within the last two years of the study period. As this number will probably increase in the future, attention should be paid to the correct choice of antibiotic agent.

Early discontinuation of treatment can lead to disease relapse; among improvement of symptoms, the duration of treatment depends on follow-up imaging. Which imaging modality is most useful to assess treatment efficacy is still a matter of discussion since conventional methods do not properly represent disease activity. At least one CT scan was performed in all patients of the current study. The most common radiological findings were soft tissue changes and bone erosion in approximately 50% of patients. Follow-up scans revealed similar results compared to first scans and failed to reveal disease progression or regression in up to 80% after four to nine months. Within the first two months after the initial CT scan, the highest progression rate was found. Nevertheless, CT is the most commonly used imaging method to diagnose and monitor SBO [7], as also underlined in this study. There is no doubt, that the assessment of bone erosion and demineralization can be shown best on CT imaging. However, initial findings in SBO can be non-specific and bone remineralization occurs late after disease resolution [22, 26]. Consequently, this modality seems less reliable to monitor treatment response.

Interestingly, an additional MRI was not performed on every patient of the present study cohort. A second MRI scan was performed in 40% after 2.7 months on average. The second scan revealed disease progression in 50%, whereas one out of four showed regression or no change compared to the first scan. Follow-up MRI scans revealed a greater proportion of disease progression compared to CT scans. Keeping the radiation burden in mind, MRI seems to be more suitable compared to CT for follow-up. However, an abnormal bone marrow signal on MRI can be persistent for a up to 12 months after successful treatment [27]. In order to overcome the drawbacks of conventional radiologic examinations in SBO, nuclear medicine imaging methods can aid the diagnosis and monitoring of therapy [9]. Only 15 nuclear medicine imaging examinations were performed in a third of the included patients. Due to reduced availability, examinations were performed after standard imaging methods were carried out and an enhanced tracer uptake was found in almost all cases. Unfortunately, no follow-up examination was performed on a regular basis so that we cannot compare the results properly. Tc-99m-MDP was the most frequently used tracer in bone scintigraphy and was combined with SPECT in most cases. Even a 10% rise in osteoblastic activity can be detected with this tracer and is therefore useful in the beginning of the disease [28, 29]. In contrast, Tc-99 uptake is also increased in post-operative and malignant conditions and can still be present in disease resolution [26]. Therefore, it is less suitable for follow-up. Out of the four Tc-99 labelled leucocyte scans, two were found to be normal although clear deterioration was found on conventional imaging modalities. Besides the inability to detect low-grade infectious foci, Tc-99 labelled leucocytes are more expensive and the diagnostic value is questionable. Beta-emitting tracers like FDG enable the detection of ongoing neutrophil activity in metabolically active tissue and therefore, can detect inflammation. FDG has been recommended as first choice in diagnosis and for decision-making when to stop treatment [9, 30]. In the present study, FDG was used in PET-CT or PET-MRI in only three cases. One patient had recurrent disease and underwent PET-CT 6 months after the first admission, which resulted in the continuation of intravenous antibiotics for another four weeks. Unfortunately, this patient had his fourth relapse nine months after first admission and died due to progressive disease. Two other patients were also monitored with PET-CT or PET-MRI and treatment was continued because of increased tracer-uptake. Nowadays, the availability of hybrid techniques such as PET-MRI is poor but there is good potential for disease assessment and treatment monitoring in the future.

## Conclusion

Skull base osteomyelitis (SBO) is a life-threatening disease with a high complication rate including persistent cranial nerve palsy and death. Unspecific headache or otalgia, even with normal findings in otoscopy, should raise the possibility of SBO in patients with diabetes or immunosuppression. Early microbiologic swabs are crucial to identify the pathogen before antibiotic treatment is administered. As no single imaging method is able to assess the full extent of SBO, a combination of anatomical and nuclear medicine imaging methods should be considered for diagnosis. Based on our data, follow-up imaging should be performed not as early as two months after the initial scan if there is no clinical evidence for disease progression. MRI should be the preferred modality rather than CT. Disease monitoring and consequently the time when treatment can be stopped is probably best performed by means of functional imaging.
